# Effects of 24 h Compression Interventions with Different Garments on Recovery Markers during Running

**DOI:** 10.3390/life11090905

**Published:** 2021-08-30

**Authors:** Jean Carvalho, Marcos Roberto Kunzler, Jose Ignacio Priego-Quesada, Inmaculada Aparicio, Pedro Pérez-Soriano, Álvaro Sosa Machado, Felipe Pivetta Carpes

**Affiliations:** 1 Applied Neuromechanics Research Group, Laboratory of Neuromechanics, Federal University of Pampa, Uruguaiana 97500-970, RS, Brazil; jean.carvalho@acad.ufsm.br (J.C.); marcoskunzler@unipampa.edu.br (M.R.K.); alvaromachado2005@gmail.com (Á.S.M.); 2 Research Group in Sports Biomechanics (GIBD), Department of Physical Education and Sports, University of Valencia, 46010 Valencia, Spain; j.ignacio.priego@uv.es (J.I.P.-Q.); i.aparicio.gibd@gmail.com (I.A.); pedro.perez-soriano@uv.es (P.P.-S.); 3 Biophysics and Medical Physics Group, Department of Physiology, University of Valencia, 46010 Valencia, Spain; 4 AITEX (Textil Research Institute), 46010 Alcoy, Spain

**Keywords:** kinematics, compressive garment, skin temperature, exercise recovery, menthol, camphor

## Abstract

Compression and temperature manipulation are discussed as strategies to improve performance markers and recovery in sports. Here, we investigate the effects of compression stockings made with fabric, either combined or not with heating and cooling substances, on variables related to running performance and recovery. Ten trained runners (mean ± standard deviation age 45 ± 9 years old, body mass 69 ± 7 kg, height 166 ± 4 cm) with no experience of using compression garments performed an intense running session of 10 km, then wore a stocking for 24 h (randomized; without compression, compression, compression with camphor, and compression with menthol), and were evaluated on the following day, after running 5 km. The different types of compression stockings used 24 h before exercise did not affect running kinematics (*p* > 0.14), skin temperature (*p* > 0.05), heart rate (*p* > 0.12; mean value of maximal heart rate 156 bpm), comfort perception (*p* = 0.13; mean value of 7/10 points), or perception of recovery (*p* = 0.13; mean value of 7/10 points). In general, there were no effects of 24 h pre-exercise lower leg compression, including those treated with menthol and camphor applications on running kinematics, skin temperature, heart rate, or recovery perception in athletes undertaking consecutive running exercises.

## 1. Introduction

Athletes are interested in compression garments to improve athletic capacity and exercise recovery, but there is still a lack of evidence regarding their biomechanics and comforting effects. While compression can affect muscle soreness, muscle damage and inflammatory markers, performance, fatigue, and thermoregulation markers are usually unaltered [1]. Biomechanic parameters, and more interestingly, athletes’ beliefs, underline the importance of including a placebo when assessing compression effects [2].

Previous studies investigating the lower extremity biomechanics in participants subject to compression showed controversial results. Hip and thigh compression may influence the performance of jump landing tasks due to decreased dynamic valgus in the landing [3] and reduced sagittal plane range of motion for the hip joint [4]. Such results have been reported as dependent on reduced muscle oscillation, improved force production [5], and joint stiffness [6]. More importantly, previous studies suggested a compression effect on important kinematics characteristics of articular movements. Theoretically, these effects could also be interpreted as useful for injury prevention.

The effects of compression on running performance parameters have attracted the interest of scientists, but knowledge about their effects on running kinematics, for example, is limited. Moreover, evidence to support the claim of below-knee compression garments in improving recovery in runners is also limited [2]. The mechanisms explaining the effects of compression remain unclear, mainly being discussed as dependent on changes in blood flow and oxygen delivery [7], reduced muscle oscillation and muscle activation [8], and the influence of an athlete’s beliefs about the benefits of compression [2].

For two consecutive 5 km running sessions with a 1 h recovery period, wearing compression stockings in between did not influence performance time and rate of fatigue. When the same participants were grouped according to their perception of the efficacy of compression stockings (being classified as believers or not), the runners with a stronger belief in the effects of compression showed improved running performance after 1 h compression compared to those with a neutral or negative perception of compression effects [9]. When 4 h compression was administrated during and after intense running, there were beneficial trends in performance and reduced muscle soreness in the following 24 h post-exercise period [10]. Soccer athletes also reported reduced perception of muscle soreness under conditions of cumulative exercise and use of compression garments [11]. The effects of a more extended period of compression are still debatable. During recovery, positive effects of 24 h compression were found in cyclists completing two consecutive time trials [12]. They achieved a 3.3% improvement in power output after compression without changes in the rate of perceived effort and oxygen uptake [12]. Similar outcomes were described for shorter sessions of high-intensity cycling, in which compression was applied during the recovery period [13].

Considering that different studies report different responses to compressive garments, outcomes are often discussed as dependent on blood flow and oxygenation, and therefore body temperature could play a role. For example, manipulating temperature is a physiological strategy for improving muscle contraction, which helps to explain why a warming-up period before exercise can benefit performance [14]. The use of a garment promoting heating could reinforce this mechanism. Before and during warming-up, skin cooling also seems to improve exercise performance [15]. Ice ingestion, for instance, has been used as an internal cooling strategy to improve running performance parameters [16].

It is challenging to combine temperature manipulations while delivering compression. One option is to use chemical compounds combined with the textile material. A compression fabric with menthol application promotes the perception of freshness and reduces heating discomfort [17]. On the other hand, camphor initially induces a perception of coolness that changes to heat stimulation, so presenting effects on blood circulation and performance, similar to that of the menthol application [18]. Therefore, compression garments combined with these compounds could promote an additional stimulus by eliciting changes in the local temperature of the skin and muscles. In this study, we determine the effects of pre-exercise lower leg compression, either combined or not with heating and cooling substances, on variables related to running recovery when performing intense exercise on two consecutive days. We hypothesized that different compression garments administrated during the 24 h recovery period, including those with the application of chemical compounds such as menthol and camphor, could positively influence biomechanics’ characteristics of running technique, heart rate, and recovery markers on consecutive days of running. Such a positive influence would translate into similar or better outcomes when the compression conditions were compared with the control condition.

## 2. Materials and Methods

### 2.1. Participants and Experimental Design

The research was advertised in local running clubs, and experienced runners were invited to join the experiment. They were invited to visit the laboratory eight times within 12 weeks when no competition was scheduled. The eight visits always included a block of two consecutive days of indoor running, one for each of the control or compression garment conditions. We were able to include ten experienced male competitive runners in the study. They had mean ± standard deviation age 45 ± 9 years old, body mass 69 ± 7 kg, height 166 ± 4 cm, and have frequently been training over the past 14 ± 13 years, with a 5 km personal record of 20:13 ± 3:54 min (fastest participant: 18:05 min; slowest participant: 23:00 min). All participants were members of running clubs from the local community, had been training and running for at least one year uninterruptedly, had no history of injury or pain, had participated in amateur competitive events with distances from 5 to 42 km, and did not have any previous experience of using compression. Participants not completing all the evaluation sessions in the expected period of 12 weeks were excluded. The participants had never worn leg compression garments before and did not classify themselves as believers in compression effects during an interview, which included questions such as “do you know what compression garments are?” and “do you believe they can improve performance during running?” The local institution ethics committee approved this research, and all participants signed a consent term. All procedures complied with the Helsinki declaration.

**Figure 1:**
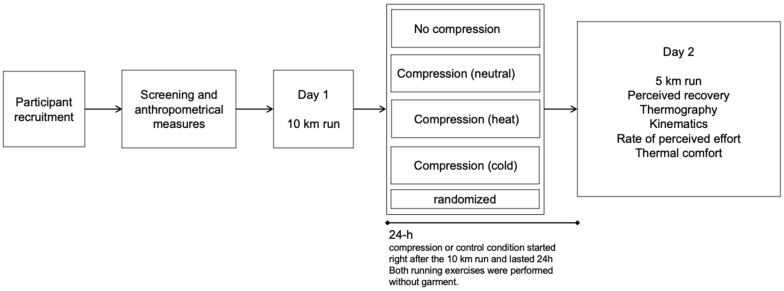
Experimental design. All participants ran day 1 without the control or compressive garment. After the running exercise, they wore the garments for 24 h before returning to the laboratory for assessment on day 2. As we tested one control and three compression conditions, each participant repeated the protocol four times.

Compression and control garment conditions were randomized. On day 1, participants ran 10 km at their competitive pace. On day 2, they ran 5 km at the same speed as day 1, and the data were collected. Garments were provided after the end of exercise on day 1 and worn continuously for the following 24 h. Each garment condition was evaluated at least one week apart, and participants always used the same shoes. Running was performed without the garments. Figure 1 shows the experimental design.

### 2.2. Compression Stockings

Four different garments were considered. They were all long socks from the knee popliteal line to the foot. The compression garments were stockings made of 10% polyamide, 75% polyester, and 15% elastomer. Three compression models were used as the standard and two others that included camphor and menthol to induce the perception of hot and cold. The garments’ compression was gradual, with an average of 21–24 mmHg pressure reported by the manufacturer. A finishing textile process in a padding machine applied camphor and menthol; the concentration of the solution was: 7% of the respective substance (camphor or menthol), 3% of polyvinyl pyrrolidone (PVP), 35% of ethanol, and 55% of water. After impregnation, the garments were dried for polymerization. The control stocking was a model of the same color and size but made of 100% cotton. Cotton socks are standard for a runner, and we therefore considered it as a control. The purpose of compression garments is to promote greater compression than cotton socks. All the stockings were brand new, from the same manufacturer, and had identical designs and appearance. Sizes were adjusted for each participant considering leg circumference and according to the manufacturer’s sizing chart.

### 2.3. Running Protocols

Running was performed on a motorized treadmill with control for speed and inclination set at 1% (Gait Trainer 3, Biodex Inc., Shirley, NY, USA). Running was performed at a competitive race pace corresponding to 90% of the best 10 km personal record [9]. A warm-up was conducted before the protocol by walking 3 min at 1.4 m/s, followed by 3 min running at 2.2 m/s. The average running speed for the running sessions was 3.5 ± 0.2 m/s. After completing 10 km, the athletes received a garment and wore it for 24 h until they returned to the lab for assessment on day 2. They were requested to take the garment off only for showering and not to perform any recovery strategy. On day 2, the 5 km running protocol was performed at the same individual speed as day 1, without garments, and kinematic, perception, and skin temperature measurements were undertaken.

### 2.4. Kinematic Assessment

Trunk, hip, knee, and ankle sagittal plane angles determined at the foot strike instant, step frequency, and step length were determined by 2D video analysis [19]. Measurements were taken at the 1st, 3rd, and 5th kilometers of the 5 km run on day 2 with a video camera placed aside the treadmill with the lens perpendicular to the plane of movement. The camera was positioned on a tripod of 90 cm height and 3 m away from the volume of movement. Movements were recorded at 30 Hz and further de-interlaced to 60 Hz using a motion analysis tool (Kinovea 0.8.15-https://kinovea.org, accessed on 6 July 2021). Spherical reflexive markers of 14 mm diameter were placed on the shoe at the fifth metatarsal, lateral malleolus, lateral knee epicondyle, great trochanter, and acromion on the right side of the body. Joint angles were defined in the sagittal at the foot strike as illustrated in Figure 3 (trunk angle defined according to the vertical axis, hip angle defined according to the horizontal axis, relative angles for knee and ankle, see Figure 3). Ten complete strides were analyzed for each kilometer considered [19]. All participants presented a rear strike landing pattern.

### 2.5. Analysis of Heart Rate, Comfort, and Perceived Recovery

On day 2, participants were questioned about their perception of recovery regarding the exercise performed the day before. The perception of recovery was assessed considering a visual scale ranging from 0 (very poorly recovered/extremely tired) to 10 (very well recovered/highly energetic) [20]. For both days, heart rate during running was monitored every second using a heart monitor (F50, Polar Electro Oy, Espoo, Uusimaa, Finland), and the data were averaged over each minute of the running session, excluding the warm-up period. The highest heart rate (HR) value was then considered for the statistics. The perception of comfort in wearing the stockings was assessed through an adapted analog visual scale [21] when participants arrived at the laboratory on day 2. The scale ranged from uncomfortable (0 cm) to the most comfortable condition imaginable (10 cm). The comfort parameters analyzed were general comfort in wearing the garment, comfort perception as regards the feel on touching the fabric material, perceived calf compression, humidity, and perceived temperature. An average score was determined to represent the overall comfort. We preferred to use the heart rate instead of the rate of perceived effort (Borg Scale) to describe exercise intensity given that two other visual analog scales were already being used to monitor recovery and comfort. Heart rate, comfort, and perceived recovery were averaged across the participants for assessing the different garments.

### 2.6. Skin Temperature

**Figure 2:**
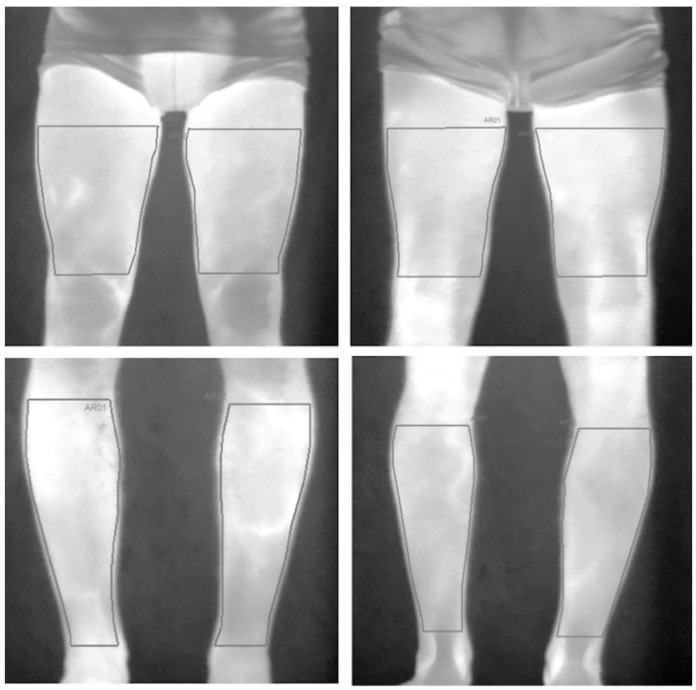
Regions of interest (ROI) defined for the infrared thermography data analysis. The top images represent the anterior (**left**) and posterior (**right**) view of the thigh, and the bottom images represent the anterior (**left**) and posterior (**right**) view of the lower leg.

Skin temperature was determined before and immediately after each running session using a thermal infrared camera (E-60, 320 × 240 pixels, FLIR Systems Inc., Wilsonville, OR, USA) with Noise Equivalent Temperature Difference (NETD) < 0.05 °C, and measurement uncertainty of ±2 °C or 2%. To record the images, the camera was positioned 1 m away from the participant and with the lens aligned perpendicular to the body region of interest (ROI) and turned on 10 min before taking the images to ensure its stabilization. The images were recorded while the participant was standing up wearing underpants after a room thermal adaptation of 10 min. An anti-reflective panel was placed behind the participant to minimize the effects of infrared radiation reflected from the wall. All recommended procedures regarding environmental conditions (e.g., room temperature) and participant preparation regarding drinking alcohol or caffeine, smoking, using cosmetics, having large meals, using ointments, sunbathing, engaging in physical activity, and undergoing physiotherapy treatments were followed to minimize influence factors. The TISEM checklist was completed to certify that all the important protocol and thermographical analysis aspects were attended to [22]. Four ROIs were defined (anterior and posterior thigh and lower leg) in both lower limbs (Figure 2).

Each ROI was defined with a similar area for all participants. Temperature data(mean, maximum, minimum, and variation (difference post-pre exercise)) were analyzed using commercial software (Thermacam Researcher Pro 2.10 software, FLIR, Wilsonville, OR, USA), taking an emissivity factor of 0.98 [23] into account. The environmental conditions during the measurements were a temperature of 23 (1) °C and relative air humidity of 60 (10)%.

### 2.7. Statistical Analysis

Data from the different conditions are reported as mean ± standard deviation. The normality of data distribution was checked using the Shapiro–Wilk test (*p* > 0.05). Repeated-measures ANOVA with two factors (measurement moment and garment condition) was applied for kinematic (stride length, stride rate, and the trunk, hip, knee, and ankle angles) and comfort parameters. Resting heart rate, maximum heart rate, and recovery rate were assessed with repeated-measures ANOVA with one factor (garment condition). For leg perimeter and skin temperature parameters, repeated-measures ANOVA with three factors (measurement moment, condition stocking, and lower limb dominance) was applied. When significance was found, the Bonferroni post hoc test was applied to identify the differences. The significance level was set as 0.05.

## 3. Results

The participants completed the 10 km run in 49:15 ± 3 min and the 5 km in 24:50 ± 1:30 min. The running speed was 3.5 ± 0.2 m/s, with an inter-participant variation coefficient (standard deviation to mean ratio) of 6.46%.

### 3.1. Effect of Compression Garments on Kinematics Parameters

**Figure 3:**
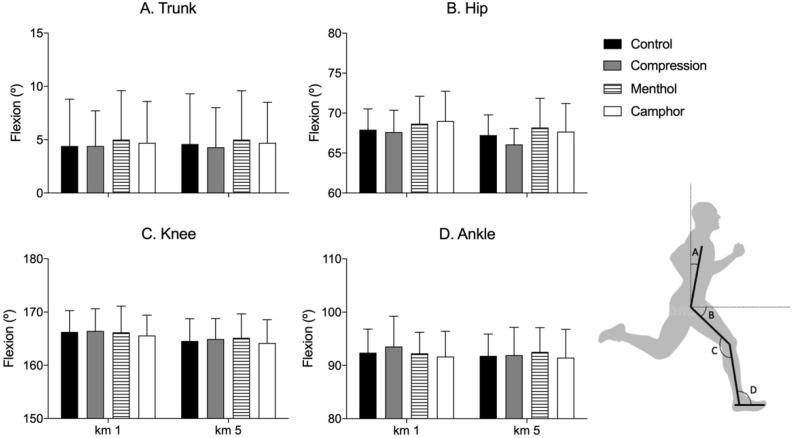
Mean (bars) and standard deviation (vertical lines) results of the sagittal plane kinematics found at different moments of the 5 km run (1st and 5th km) in the compression and control garment conditions. No differences were observed between garment conditions. The representation of the body angles indicates the trunk (**A**), hip (**B**), knee (**C**), and ankle (**D**) angles determined in the sagittal plane at the foot strike instant.

The stride length and stride rate did not differ between the different garment conditions (stride length: main effect of stocking conditions *p* = 0.15; main effect of moment *p* = 0.24, stocking * moment *p* = 0.37; stride rate: stocking condition *p* = 0.16, moment *p* = 0.26, and stocking condition * moment *p* = 0.37). The mean stride length and stride rate were 0.79 ± 0.06 m and 4.3 ± 0.2 stride/s, respectively. No differences in angular kinematics were observed between compression and control conditions (Figure 3; trunk sagittal plane angle: stocking condition *p* = 0.82, moment *p* = 0.94, and stocking condition * moment *p* = 0.90; hip sagittal plane angle: stocking condition *p* = 0.18, moment *p* < 0.01, and stocking condition * moment *p* = 0.61; knee sagittal plane angle: stocking condition *p* = 0.53, moment *p* < 0.01, and stocking condition * moment *p* = 0.91; ankle sagittal plane angle: stocking condition *p* = 0.82, moment *p* = 0.19, and stocking condition * moment *p* = 0.38).

### 3.2. Effect of Compression Garments on Skin Temperature

The main effect of each factor and its interactions with the other factors of the repeated-measures ANOVAS models are shown in Table 1. The main effect of the lower limb and its interaction with the other factors was not significant for any of the ROIs and parameters assessed (*p* > 0.05). Although the garment conditions main effect was significant for anterior thigh, when considering the measurement of mean skin temperature and maximum skin temperature and its interaction with measurement moment for the anterior leg of minimum temperature, the post-hoc analysis did not reveal differences between garment conditions (*p* > 0.05). Table 2 shows the skin temperature parameters (mean, maximum, minimum, and variation) of the different ROIs with the mean data of both lower limbs.

### 3.3. Effect of Compression Garments on Heart Rate, Comfort, and Recovery

**Figure 4:**
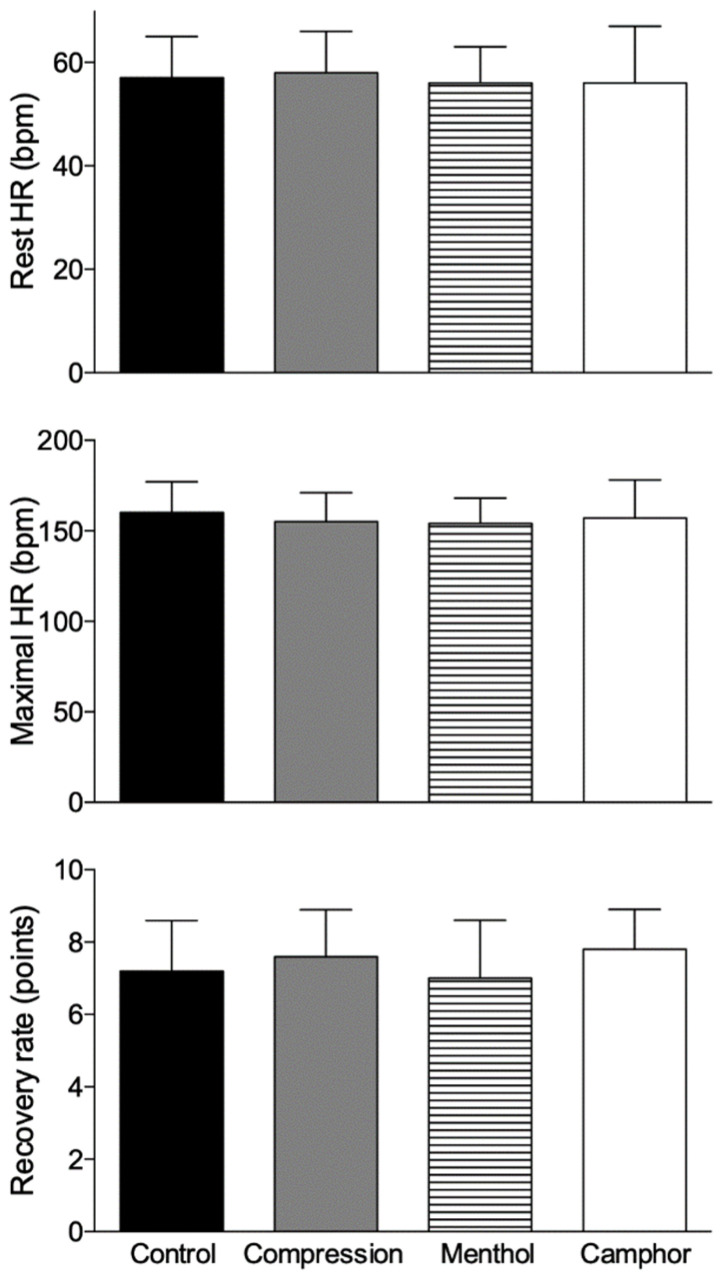
Mean (bars) and standard deviation (vertical lines) results of the rest (before exercise) and maximal heart rate (during exercise) and the perceived recovery rate before exercise on day 2. No difference was found between the garment conditions.

No differences were observed for the rest (*p* = 0.81) and maximal heart rate (*p* = 0.13) between the stocking conditions (Figure 4). For all the comfort parameters analyzed, in all conditions and moments, overall comfort was reported on average at degree 6 of 10 possible points with an average value of 7 (1) points. It did not differ between the garment conditions (*p* = 0.13). Perception of recovery evaluated on day 2 did not differ between the garment conditions (*p* = 0.86), presenting an average value of 7.4 (0.4) points (Figure 4).

## 4. Discussion

Our study aimed to determine the effects of 24 h lower leg compression using different compression garments, including those inducing perceptions of hot and cold, on aspects of running performance by considering the kinematic assessment of technique, intensity, comfort, and recovery following consecutive running exercises. Our main findings were that the different types of compression, including chemical compounds, such as menthol and camphor, or not on the lower leg did not affect the parameters evaluated. Importantly, rest and maximal heart rate did not differ between experimental conditions, suggesting that a similar intensity was performed for all tests. Under the test conditions performed here, compression did not present any perceived advantage for the runners.

One could argue that the magnitude of compression provided by the garment can influence the results. However, a previous study showed that compression effects were not associated with the magnitude of pressure applied [7]. The pressure applied by sports compression garments is significantly affected by garment type, size, and body posture [24]. To minimize these effects and limitations, we also tested a control garment (without compression) of similar size and color characteristics as the compression stockings and matched the size to the size recommended to each participant. The results for the control stocking did not differ from those for the compression stockings.

In a previous study, compression administrated to the hip and thigh elicited changes in frontal plane kinematics towards a movement pattern considered protective against knee injuries during jump landing [3]. Here, we analyzed the running exercise and found that for sagittal plane angles at the foot strike, there was no significant difference between the compression conditions and the control. This may suggest that foot landing strategies evaluated by ankle angle at the sagittal plane do not change during running sessions, which may result in minor or absent effects on running economy, according to previous research [25]. Stride frequency, associated with lower extremity loading during running [26], did not differ between the stockings conditions.

There are controversial results in the literature about the effects of compression garments on thermal stress. If compression garments increase skin temperature, this could be negative for performance due to the reduced thermal gradient between the core and the skin [27]. We did not find differences in lower leg skin temperature between the different garment conditions. The influence of the menthol and camphor applications probably wore off after a short time during the prolonged use of the garment. The influence of the menthol and camphor applications may differ by using different concentrations of the compounds [28]. Furthermore, changes in skin temperature depend on the type of exercise, environmental conditions, and/or the type of garment and its properties. While studies with upper-body compression garments observed increases in core and skin temperature during aerobic exercise [29], studies with lower-body compression did not observe any apparent effect on skin and core temperature [30]. In addition, low transpiration of the textile could result in a higher increase in the skin temperature during exercise [31]. Therefore, it could be speculated that all the garment conditions enjoyed good textile transpiration, so resulting in no differences compared to the no stocking condition. This assumption appears to be supported in our results concerning comfort.

The use of fabrics that promote thermal interactions between the stocking and the skin did not influence recovery perception and exercise intensity. Positive effects were described for menthol in thermal perception, ventilatory responses, analgesia [32], and increased localized blood flow in the skin and muscle, as too it was for camphor applications [18]. Those effects were observed in the short term after application, and, as we mentioned before, we hypothesize that they wear off shortly afterwards, given that in our experiment the garments were worn for 24 h. It is true, however, that some of the participants informally mentioned a feeling of relaxation and freshness in the lower leg while using the menthol and camphor stocking. Yet, as our results show, there were no significant effects on the parameters considered in the comfort assessment, and we did not determine for how long such a perception was present. It is also important to mention that thigh ROIs were assessed based on the results of a previous study showing higher skin temperature in these regions using compression stockings during running [33]. Our data provide additional support to the compression approach’s lack of effect in terms of time outcomes for runners, as previously described [34].

Our study has limitations. The compression garment may influence arterial blood pressure both locally and in the body, as well as the percentage of the total body water. We did not measure these parameters that influence the vein return and final cardiac output. The maximal or the resting heart rate is not the best physiological variable to assess the impact of compression on cardiovascular function. We used a 2D video for kinematics analysis, as it has been reported that 2D video analysis is accurate enough to evaluate the variables that we assessed during treadmill running [35]. The results provided are dependent on the protocol chosen. We opted for a protocol eliciting cumulative running load, and therefore the effects of more prolonged running sessions, exhaustion, or fatigue still need to be clarified. Another limitation was the inability to monitor temperature changes over the 24 h of compression, especially for the garments prepared with menthol and camphor. Finally, we must bear in mind that interpreting the comfort scale may vary with climate, season, and language.

## 5. Conclusions

In this study, we investigate the effects of compression stockings made with fabric, either combined or not with heating and cooling substances, on variables related to running performance and recovery. Overall, no effect of 24 h lower leg compression including menthol and camphor applications was found on characteristics of running performance considering kinematics, heart rate, and perceived recovery of runners performing consecutive running sessions at a competitive pace.

## Figures and Tables

**Table 1:** *p* values of the main effect and its interaction effect with each factor in the repeated-measures ANOVAS for skin temperature parameters.

ROI	Leg	Moment	Garments	Leg * Measurement	Leg * Garments	Measurement * Garments	Leg * Measurement * Garments
Mean skin temperature (°C)							
Anterior thigh	0.37	0.02	0.03	0.73	0.32	0.45	0.88
Posterior thigh	0.10	0.21	0.26	0.13	0.17	0.74	0.78
Anterior leg	0.69	<0.01	0.13	0.95	0.19	0.69	0.24
Posterior leg	0.58	0.04	0.30	0.73	0.53	0.55	0.15
Maximum skin temperature (°C)							
Anterior thigh	0.24	<0.01	0.04	0.50	0.25	0.98	0.09
Posterior thigh	0.89	<0.01	0.43	0.32	0.20	0.41	0.53
Anterior leg	0.49	<0.001	0.27	0.60	0.08	0.85	0.19
Posterior leg	0.82	<0.01	0.51	0.67	0.34	0.71	0.44
Minimum skin temperature (°C)							
Anterior thigh	0.71	0.04	0.04	0.10	0.63	0.56	0.09
Posterior thigh	0.08	0.17	0.17	0.09	0.74	0.27	0.22
Anterior leg	0.14	0.06	0.21	0.09	0.05	0.02	0.29
Posterior leg	0.62	<0.01	0.87	0.23	0.71	0.13	0.68
Δmean skin temperature (°C)							
Anterior thigh	0.73	-	0.45	-	0.88	-	-
Posterior thigh	0.13	-	0.74	-	0.78	-	-
Anterior leg	0.95	-	0.69	-	0.24	-	-
Posterior leg	0.73	-	0.55	-	0.15	-	-

* statistics for interactions.

**Table 2:** Mean (SD) of the skin temperature parameters (mean, maximum, minimum, and variation of mean temperature) of the four regions of interest (ROI) assessed before and after exercise in the four garment conditions.

ROI	Before Exercise				After Exercise			
	Control	Compression	Menthol	Camphor	Control	Compression	Menthol	Camphor
	Mean Skin Temperature (°C)							
Anterior thigh	31.6 (1.5)	30.8 (1.7)	31.3 (1.0)	32.0 (1.0)	32.7 (1.5)	32.0 (1.1)	32.6 (1.1)	32.6 (1.1)
Posterior thigh	32.1 (1.3)	31.6 (1.4)	31.8 (0.9)	32.1 (0.8)	32.5 (1.2)	32.0 (1.0)	32.4 (1.0)	32.4 (1.1)
Anterior leg	32.2 (0.9)	31.6 (1.1)	31.7 (1.0)	32.1 (0.9)	32.9 (1.0)	32.3 (0.8)	32.7 (0.6)	32.7 (0.6)
Posterior leg	31.9 (1.0)	31.5 (1.3)	31.6 (0.7)	32.0 (0.6)	32.6 (1.2)	32.1 (1.1)	32.6 (0.8)	32.5 (0.9)
	Maximum skin temperature (°C)							
Anterior thigh	33.6 (1.2)	33.0 (1.5)	33.6 (1.0)	33.6 (0.9)	35.0 (1.1)	34.3 (0.8)	34.8 (0.8)	34.8 (0.8)
Posterior thigh	33.7 (1.1)	33.4 (1.1)	33.3 (0.9)	33.6 (0.6)	34.5 (0.9)	34.1 (0.7)	34.6 (0.6)	34.4 (1.0)
Anterior leg	33.6 (0.9)	33.3 (1.1)	33.4 (1.1)	33.6 (0.9)	34.9 (0.9)	34.4 (0.7)	34.7 (0.6)	34.7 (0.6)
Posterior leg	33.2 (1.0)	33.0 (1.2)	33.1 (0.9)	33.2 (0.7)	34.3 (0.9)	33.9 (0.9)	34.3 (0.8)	34.2 (0.8)
	Minimum skin temperature (°C)							
Anterior thigh	28.9 (2.2)	28.0 (1.9)	28.5 (1.2)	29.4 (1.5)	30.0 (2.0)	29.0 (1.8)	29.9 (1.3)	29.4 (1.5)
Posterior thigh	29.4 (2.1)	28.6 (1.8)	29.2 (1.3)	29.4 (1.7)	29.7 (2.2)	28.9 (1.3)	30.0 (1.2)	29.4 (1.5)
Anterior leg	29.1 (2.1)	28.4 (1.6)	28.2 (1.8)	29.4 (1.7)	29.8 (2.5)	28.8 (1.3)	30.1 (1.1)	29.7 (1.5)
Posterior leg	29.1 (2.3)	28.9 (1.5)	28.3 (1.6)	29.2 (1.9)	30.0 (2.4)	29.8 (1.5)	30.2 (1.8)	30.0 (1.7)
	Δ mean skin temperature (°C)							
	Control		Compression		Menthol		Camphor	
Anterior thigh	1.1 (1.7)		1.1 (1.5)		1.3 (1.2)		0.6 (1.2)	
Posterior thigh	0.4 (1.1)		0.3 (1.2)		0.6 (1.1)		0.2 (1.3)	
Anterior leg	0.7 (1.0)		0.6 (0.8)		1.0 (0.8)		0.6 (1.1)	
Posterior leg	0.7 (1.1)		0.5 (1.2)		0.9 (0.9)		0.4 (1.2)	

## Data Availability

Data can be made available by the author upon request.
